# Semi-Crystalline Polymers Applied to Taylor Impact Test: Constitutive, Experimental and FEM Analysis

**DOI:** 10.3390/polym12071615

**Published:** 2020-07-21

**Authors:** Lizhi Xu, Chun Cheng, Chengxin Du, Zhaoxiu Jiang, Zhonghua Du, Guangfa Gao

**Affiliations:** School of Mechanical Engineering, Nanjing University of Science and Technology, Nanjing 210094, China; xulznjust@163.com (L.X.); xiangchun893@163.com (C.C.); duchengxin4324@163.com (C.D.); A1695925277@163.com (Z.J.); duzhonghua@aliyun.com (Z.D.)

**Keywords:** semi-crystalline polymers, constitutive model, Taylor impact, LS-DYNA, finite element simulation

## Abstract

Based on mechanical properties of Polyamide 66 (PA66) under complex loading conditions, a Drucker–Prager yield criterion was employed to characterize its yield behavior. Then, a one-dimensional model, which contains a viscoelastic regime and a viscoplastic regime, was introduced and converted into a three-dimensional constitutive model. The three-dimensional model was implemented into a LS-DYNA software, which was used to predict the dynamic response of PA66 under Taylor impact conditions, whose corresponding tests were conducted by gas gun and recorded by high-speed camera. By contrasting the simulation results and these of the corresponding tests, the deformed shapes including the residual length, the maximum diameter and the shape of the mushroom head of the PA66 bars were found to be similar to these obtained from the tests, which verified the accuracy of the three-dimensional constitutive model, and proved that the model was able to be applied to high-rate impact loading conditions.

## 1. Introduction

Semi-crystalline polymer materials possess low density and good toughness, so they are widely applied in many aspects, such as bullet cores and electromotive tools. It is well-known that these bullet cores and electromotive tools are always subjected to extreme loadings. Therefore, it is necessary to study their mechanical properties under complex loading conditions, and the research results are able to guide us to use these polymers safely. Until now, tensile and compressive behaviour of semi-crystalline polymers has been studied under complex conditions (various temperatures and strain rates) [1,2,3]. For example, dynamic properties of polyethylene were studied over high strain rates by Xu et al. [4]. Based on the dynamic mechanical test results, it was revealed that LDPE possesses a much smaller yield stress and failure strain than extruded UHMWPE. Omar et al. [5] studied the influence of strain-rate on three kinds of polymeric materials: polycarbonate (PC), polypropylene (PP) and polyethylene (PE). The test results indicated that the mechanical properties of the compression modulus and compressive strength increased with the increase of strain rates. Duan et al. [6] proposed a uniform phenomenological constitutive model for semi-crystalline polymers on basis of four models: Johnson Cook model, G’ Sell Jonas model, Matsuoka model, and Brooks model. Based on quasi-static and dynamic compressive and tensile tests of Nylon 6, Pouriayevali et al. [7] proposed a constitutive model to describe the dynamic properties of the semi-crystalline polymer (Nylon 6 as a representative).

At present, the research on yield criterion of polymers mainly focuses on the following aspects: (1) Bowden and Jukes [8] introduced pressure terms into Tresca criterion and Von Mises criterion (Table 1, R1 and R2), and they considered that they satisfy a linear relationship between the maximum shear stress or second invariant of deviatoric stress (*J*_2_) and hydrostatic pressure (*I*_1_/3). Experimental results showed that polymethyl methacrylate (PMMA) and polystyrene (PS) materials satisfy the modified Tresca yield criterion and modified Von mises yield criterion, respectively. (2) Raghava et al. [9] also modified the Von mises yield criterion (Table 1, R3) by linearly relating the second invariant of deviatoric stress (*J*_2_) to hydrostatic pressure (*I*_1_/3), but the linear parameters are determined by compressive strength and tensile strength of polymers. The modified yield criterion was verified by PC and polyvinylchloride (PVC) materials. (3) Silano et al. [10] established a unified form of power polynomial function (Table 1, R4), which accounts for hydrostatic pressure dependency of yield strength. It is clear that this equation is reduced to Von Mises and Drucker Prager yield criterion when *N* = 0 and *N* = 1, respectively. The yield behaviour of polyoxymethylene (POM) and polypropylene (PP) were predicted by Pae et al. [11] using this equation with *N* = 1 and *N* = 2, respectively. Based on previous work, Ghorbel [12] established a generalized yield criterion (Table 1, R5) which is introduced the third invariant of deviatoric stress (*J*_3_) to yield criterion. Farrokh [13] built a yield criterion dependent on strain rate (Table 1, R6) for isotropic polymers at different rates.

Previous numerous studies provide sufficient help in understanding the mechanical properties of semi-crystalline polymers. A one-dimensional constitutive model has been proposed to predict the mechanical behaviour of semi-crystalline polymers by our team [14]. The purpose of this paper is to convert the one-dimensional model into three-dimensional constitutive model, to demonstrate the accuracy of the three-dimensional model and to realize application of the constitutive model in LS-DYNA software. Therefore, based on the mechanical properties of PA66 under complex loading conditions, a Drucker Prager yield criterion was used to characterize its yield behaviour. Moreover, compression tests were performed by using SHPB equipment to confirm the unknown parameters of the constitutive model. Serval sets of Taylor impact tests were performed to verify the accuracy of the three-dimensional constitutive model, and proved that the model was able to be applied to high-rate impact loading conditions.

## 2. Description of the Constitutive Model

**Figure 1:**
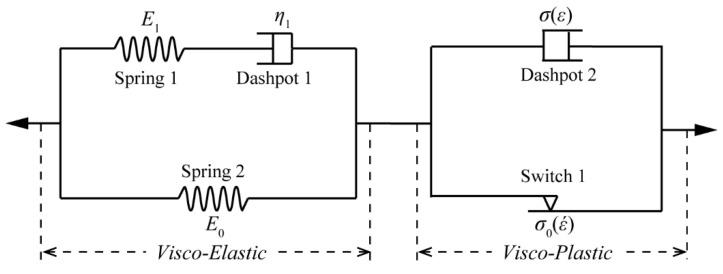
Structure of the one-dimensional constitutive model.

In previous study [14], a one-dimensional constitutive model was proposed to characterize dynamic properties of semi-crystalline polymers. Figure 1 displays a structure of the model, which consists of a viscoelastic and a viscoplastic phase. For the viscoelastic phase, it includes two elastic springs and a dashpot. One elastic spring and the dashpot are in series, and then they are together parallel to the other spring to constitute a standard Kelvin model [15]. The standard Kelvin model is able to characterize the nonlinear (stress-strain relationship) and viscoelastic properties of semi-crystalline polymers. For the viscoplastic phase, a stress-threshold switch, related to strain rate, is used to describe the yield stress of the material. It means that the stress-threshold switch will be turned on, when material stress state is greater than its yield stress. Moreover, a non-linear dashpot, in parallel to the stress-threshold switch, is going to work and describe the plastic flow deformation, after the stress-threshold switch is activated.

### 2.1. Description of the Viscoelastic Part

Stress-strain relationship of the standard Kelvin model is expressed as:

(1) $$\sigma \left(\varepsilon ,\dot{\varepsilon }\right)=E_{0}\varepsilon +E_{1}\tau_{s}\dot{\varepsilon }\left(1-\exp\left(-\frac{\varepsilon }{\tau_{s}\dot{\varepsilon }}\right)\right)$$

where $E_{0}$, $E_{1}$, $\dot{\varepsilon }$, $\tau_{s}=\eta_{1}/E_{1}$ and $\eta_{1}$ are the equilibrium elastic modulus, transient elastic modulus, constant strain rate, relaxation time and viscosity coefficient, respectively. However, the model is unable to describe the strain-rate response, since the coefficients $E_{1}$ and $\tau_{s}$ are constant. Thus, the constitutive Kelvin model was modified as follows: the coefficients $E_{1}$ and $\tau_{s}$ are assumed to be a function of strain rate, while the coefficient $E_{0}$ is considered to be an elastic modulus at quasi-static condition. Under different strain rates, it means that the elastic modulus of material equals the sum of $E_{0}$ and $E_{1}$, and Equation (1) was modified as:

(2) $$\sigma \left(\varepsilon ,\dot{\varepsilon }\right)=E_{0}\varepsilon +E_{1}\left(\dot{\varepsilon }\right)\tau_{s}\left(\dot{\varepsilon }\right)\left(1-\exp\left(-\frac{\varepsilon }{\tau_{s}\left(\dot{\varepsilon }\right)}\right)\right)$$

The expression of the relaxation time $\tau_{s}$ and modulus $E_{1}$ were established by Yu et al. [16], who also considered the influence of temperature. In this study, the properties of semi-crystalline polymers were studied at room temperature, so the expressions of $\tau_{s}$ and $E_{1}$ were constructed as $\log\tau_{s}\left(\dot{\varepsilon }\right)=\log\alpha -\beta \log\left(\dot{\varepsilon }/{\dot{\varepsilon }}_{0}\right)$ and $E_{1}\left(\dot{\varepsilon }\right)=p\left[\exp{\left(\log\dot{\varepsilon }/{\dot{\varepsilon }}_{0}\right)}^{q}-1\right]$, respectively. Where α, β, p and q are material parameters, and ${\dot{\varepsilon }}_{0}$ is the reference strain rate.

### 2.2. Description of the Viscoplastic Part

A yield stress model was built to realize the role of the stress-threshold switch. Eyring theory, which considers the influence of temperature and strain rate, was extensively used to exhibit the yield strength of polymers [17]. Since the temperature is unconsidered in this study, the Eyring theory model was simplified, and a dynamic increase factor (DIF, it is defined as the ratio of dynamic yield stress to reference yield stress) was introduced to exhibit the yield strength of semi-crystalline polymers as follows:

(3) $$\mathrm{DIF}=\frac{\sigma_{d}}{\sigma_{s}}=1+A\sinh^{-1}{\left(\frac{{\dot{\varepsilon }}^{*}}{B}\right)}^{1/\gamma }$$

where $\sigma_{d}$ is the dynamic yield stress, $\sigma_{s}$ is the yield stress under reference strain rate, A, B, γ are material parameters, ${\dot{\varepsilon }}^{*}=\left(\dot{\varepsilon }/{\dot{\varepsilon }}_{0}\right)$ is dimensionless strain rate, and ${\dot{\varepsilon }}_{0}$ is a reference strain rate.

For the plastic behaviour of semi-crystalline polymers, it displays a plastic hardening behaviour, and the stress-strain relationship is normally an exponential expression, such as $\sigma \left(\varepsilon^{p}\right)=C+D{\left(\varepsilon^{p}\right)}^{n}$ (where $\varepsilon^{p}$ are the plastic strain, n is the hardening factor, C and D are material parameters). The exponential expression is widely used in the Jonson Cook model to exhibit the plastic behaviour. Therefore, in combination with Equation (3), a plastic model dependent on strain rate was established:

(4) $$\sigma \left(\varepsilon^{p}\right)=\sigma_{s}\left[1+m{\left(\varepsilon^{p}\right)}^{n}\right]\left[1+A\sinh^{-1}{\left(\frac{{\dot{\varepsilon }}^{*}}{B}\right)}^{1/\gamma }\right]$$

where m and n are material parameters.

### 2.3. Yield Criterion and Subsequent Yield Behavior

On the experimental and theoretical aspects, much work has been done to study the influence of hydrostatic pressure on yield behaviour of polymers [13]. To characterize the yield behaviour of polymers, Bowden and Jukes [8] modified the Tresca and Mises yield criterions by introducing hydrostatic pressure item. To build a generalized yield criterion accounting of shear banding along with hydrostatic pressure dependency, Ghorbel [12] established a yield function containing a third invariant of the deviatoric stress. The yield criterions of polymers were summarized and analysed by Jin [18]. It is found that Drucker Prager model can describe the yield surface of semi-crystalline polymers. Therefore, the Drucker Prager yield criterion was employed as:

(5) $$f=\sqrt{J_{2}}+\alpha_{1}I_{1}-\alpha_{2}=0$$

where $\alpha_{1}$ and $\alpha_{2}$ are material parameters, $I_{1}$ and $J_{2}$ are second deviatoric stress invariant and the first invariant of stress, respectively. They are expressed as $I_{1}=\sigma_{kk}$ and $J_{2}=\frac{1}{2}s_{ij}s_{ij},\;s_{ij}=\sigma_{ij}-\frac{\sigma_{kk}}{3}\sigma_{ij}$. The subscript i,j,k=1,2,3.

The isotropic hardening model can better characterize the subsequent yield behavior of semi-crystalline polymer material [18]. Material parameter $\alpha_{2}$ is related to plastic strain and strain rate, so Equation (5) can be used with the set of Equation (4) to obtain the subsequent yield surface of semi-crystalline polymers:

(6) $$\left\{ \begin{array}{l} f\left(\sigma_{ij},{\bar{\varepsilon }}^{p},{\dot{\varepsilon }}^{*}\right)=\sqrt{J_{2}}+\alpha_{1}I_{1}-\alpha_{2}\left({\bar{\varepsilon }}^{p},{\dot{\bar{\varepsilon }}}^{*}\right)=0\\ \alpha_{2}\left({\bar{\varepsilon }}^{p},{\dot{\bar{\varepsilon }}}^{*}\right)=\sigma_{s}\left(\frac{\sqrt{3}}{3}-\alpha_{1}\right)\left[1+m{\left({\bar{\varepsilon }}^{p}\right)}^{n}\right]\left[1+A\sinh^{-1}{\left(\frac{{\dot{\bar{\varepsilon }}}^{*}}{B}\right)}^{1/\gamma }\right] \end{array}\right.$$

where ${\bar{\varepsilon }}^{p}=\sqrt{\frac{2}{3}e_{ij}^{p}e_{ij}^{p}},\;e_{ij}^{p}=\varepsilon_{ij}^{p}-\frac{\varepsilon_{kk}^{p}}{3}\varepsilon_{ij}^{p}$ and ${\dot{\bar{\varepsilon }}}^{*}=\frac{\dot{\bar{\varepsilon }}}{{\dot{\bar{\varepsilon }}}_{0}}$ are equivalent plastic strain and dimensionless equivalent plastic strain rate, respectively.

## 3. Mechanical Tests

### 3.1. Low-Strain Rate Tests

Polyamide 66 was chosen as study object for this study. A cylindrical specimen of 10 mm in diameter and 20 mm in length was employed in quasi-static compression tests. The specimens were machined from an original bar of *Φ* 100 × 1000 mm. A machine (CRIMS DNS-100) was used to perform quasi-static compression tests under different strain rates (10^−2^~1s^−1^). During the uniaxial compression tests, the machine was controlled by the loading speed, which was determined by the desired rate. The machine provided the load *F* (KN) and displacement *S* (mm) data of the tests. Firstly, the *F*-*S* data was converted into engineering stress-strain curves, and then the engineering stress-strain curves were converted into the true stress-strain curves as reference [19], because the true stress-strain curves can describe the true mechanical properties.

### 3.2. High-Strain Rate Tests

**Figure 2:**
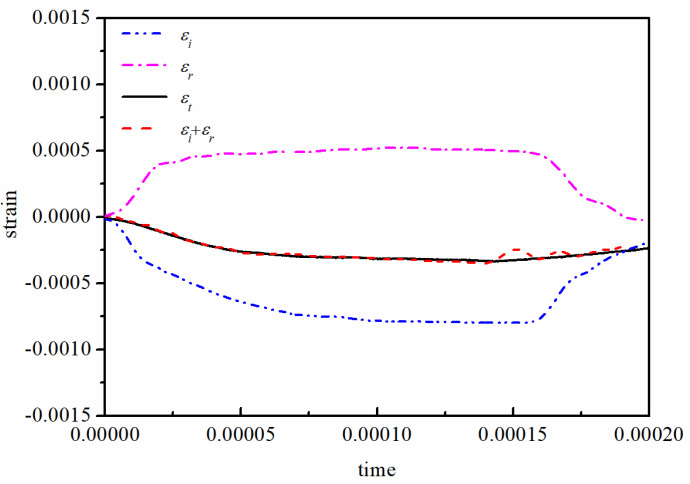
Stress equilibrium condition in specimen.

A cylindrical specimen of 10 mm in diameter and 5 mm in length was employed in dynamic compression tests [20]. These specimens were also taken from the same original bar of Φ100×1000 mm. Split Hopkinson pressure bar (SHPB) equipment of 14.5 mm diameter was employed to conduct the dynamic compression tests over high strain rates from 1000 to 4000 s^−1^. The 14.5 mm diameter SHPB equipment includes a striker bar (0.4 m in length), an incident bar (2.0 m in length), a transmission bar (1.5 m in length) and a strain testing system. The bars are made of steel whose density and elastic modulus are 7830 kg/m^3^ and 210 GPa, respectively. During the tests, copper shapers were used to adjust the stress wave, and the diameter or thickness of the copper shaper were adjusted to keep the strain-rate constant as accurately as possible according to the loading condition [21]. Based on the assumption of the stress equilibrium in the specimen and the one-dimensional wave propagation theory, the dynamic equilibrium condition was checked as shown in Figure 2. It is found that $\varepsilon_{i}+\varepsilon_{r}$ displays good agreement with $\varepsilon_{t}$. Therefore, the strain rate, stress and strain were able to be calculated by following formulas:

(7) $$\left\{ \begin{array}{l} \sigma {\left(t\right)}_{E}=E_{b}\frac{A_{s}}{A_{b}}\varepsilon_{t}(t)\\ \varepsilon {\left(t\right)}_{E}=-\frac{2C_{b}}{L_{s}}\int \varepsilon_{r}(t)dt\\ \dot{\varepsilon }{\left(t\right)}_{E}=-\frac{2C_{b}}{L_{s}}\varepsilon_{r}(t) \end{array}\right.$$

where $A_{b}$ is the cross area of the bar, $A_{s}$ is the cross area of the specimen, $L_{s}$ is the length of the specimen, $\varepsilon_{r}(t)$ is reflected strain and $\varepsilon_{t}(t)$ is the transmitted strain.

### 3.3. Test Results

**Figure 3:**
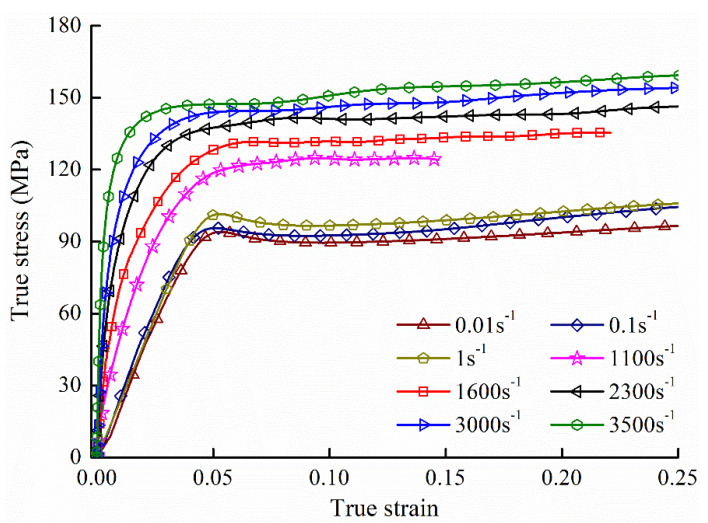
Low and high strain-rate results of PA66.

Figure 3 displays the stress-strain curves of PA66 at different strain rates. which is able to be divided into three regions: the elastic phase, the yield point and the plastic zone.

**Figure 4:**
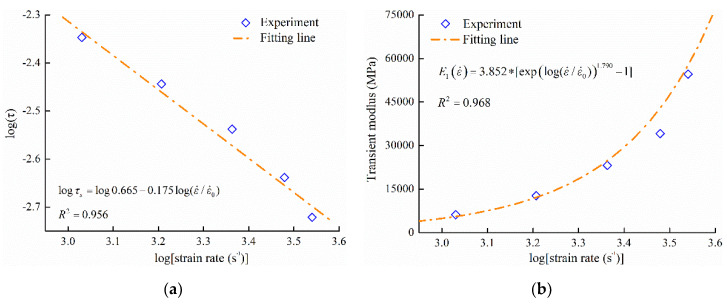
Fitting results of elastic parameters: (**a**) logarithmic forms of relaxation time; (**b**) transient modulus.

In the elastic region, PA66 behaves linear elasticity, while it behaves nonlinear elasticity at high strain rates. Based on the introduction of the constitutive model, the viscoelastic regime of the model matches these elastic properties of PA66. At low strain rates, when the strain rate increased from 0.01 s^−1^ to 1 s^−1^, the elastic modulus is almost identical. Therefore, strain rate 1 s^−1^ was considered as a reference strain rate, and the value of $E_{0}$ equals to the modulus of PA66 at strain rate 1 s^−1^. By the fitting curves of the elastic region, these parameters (α, β, p and q) of Equation (2) were confirmed, and the fitting results were shown in Figure 4a,b and listed in Table 2.

**Figure 5:**
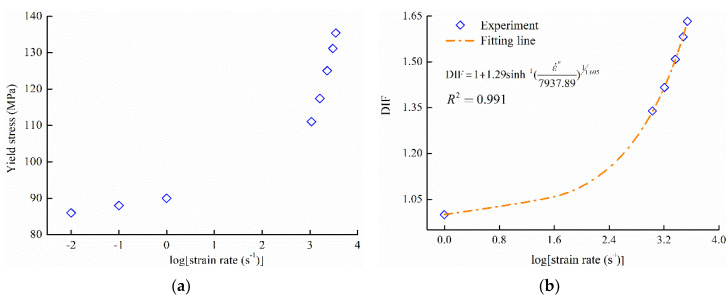
Yield stress of PA66: (**a**) test results; (**b**) fitting results.

Figure 5a shows the relationship between the yield stress and the logarithmic strain rate. It is clear that the yield stress increases by approximately 20.0 MPa, when the strain rates increase from 1000 to 4000 s^−1^. However, the yield stress increases by only 4.0 MPa at low strain rates (from 0.01 to 1 s^−1^). Therefore, the value of $\sigma_{ds}$ was also considered to be equal to the yield stress of PA66 under the condition of strain rate 1 s^−1^. By the fitting Figure 5a, these parameters (A, B, γ) of Equation (3) were confirmed, and the fitting results were shown in Figure 5b and listed in Table 2.

**Figure 6:**
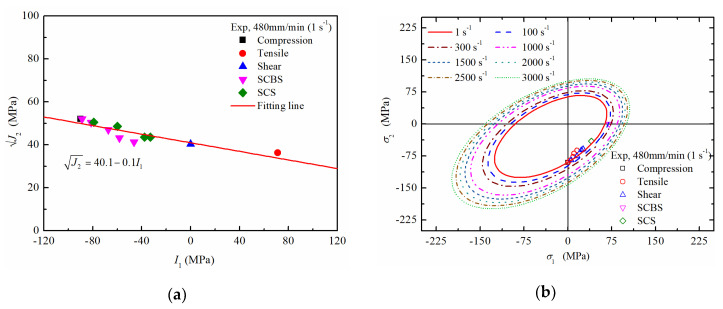

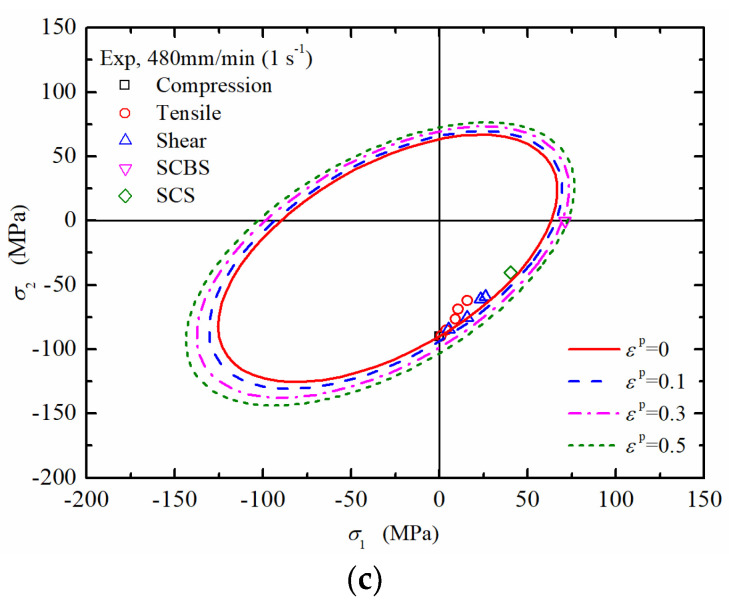
Mechanical response of PA66 under complex stress state: (**a**) material constants of the Drucker Prager yield criterion; (**b**) yield criterion of PA66 at different strain rates; (**c**) subsequent yield criterion of PA66.

The mechanical response of PA66 under complex stress state was studied by Jin [18]. He conducted compression-shear tests by two types of specimens: Shear-Compression Bar Specimen (SCBS) and Shear-Compression Specimen (SCS), and he detailly introduced the experimental method. Because the uniaxial compressive strength of the PA66 material used by Jintao is 76 MPa, the uniaxial compressive strength of the PA66 material in this paper is 90 MPa. Therefore, according to the ratio of compressive strength, the test results of SCBS and SCS were revised. The experimental data were transformed into $I_{1}\;\mathrm{vs}.\;\sqrt{J_{2}}$, shown in Figure 6a, which suggests a linear relationship between $I_{1}$ and $\sqrt{J_{2}}$. Therefore, the unknown parameters of Drucker Prager yield criterion were confirmed by fitting the data points and Equation (7), and their values were listed in Table 2. Based on Equation (6) and the initial yield criterion of PA66, the influence of strain rate on the yield criterion and subsequent yield behaviour were provided in Figure 6b,c, respectively.

## 4. Taylor Impact Research

### 4.1. Taylor Impact Test

Taylor impact tests were performed to verify the accuracy of the constitutive model of semi-crystalline polymers. The definition of the Taylor impact test is that a cylindrical specimen is accelerated to impact normally on a rigid target to produce deformation or destruction. Taylor impact tests were conducted by using a 12.0 mm diameter gas gun to launching PA66 rod specimens. The diameter and length of these samples are 12.0 mm and 48.0 mm, respectively. The impact velocities were varied from 100~170 m/s to adjust the strain-rate conditions and stress states. A Photron FASTCAM SA1.1 high-speed camera, operating at a framing rate of 40,000 frames/s and a shutter speed of 1/4000 s, was used to photographically record the cylinders shortening in length, producing a mushroom head at impact end and even failure. The specific conditions are listed in Table 3.

### 4.2. Taylor Impact Simulation

The one-dimensional constitutive model, described in Section 2, was converted into three-dimensional constitutive model, which was implemented into a LS-DYNA software through UMAT. Based on the three-dimensional finite element analysis, an incremental form of the model was deduced on the basis of radial return plasticity [22]. An explicit integration algorithm was used in LS-DYNA [23]. The maximum plastic strain and shear strain failure criterion were used to describe the failure mode of PA66. The incremental form of three-dimensional finite element model was used to describe the dynamic properties of PA66 cylindrical specimen impacting on a steel target. During the impact tests, the steel target was machined from 45CrNiMoV steel rods and heat treated to a hardness of HRC45 and the yield strength of 1420 MPa, which is much higher than PA66, so the target was viewed as a rigid target in the simulation.

**Figure 7:**
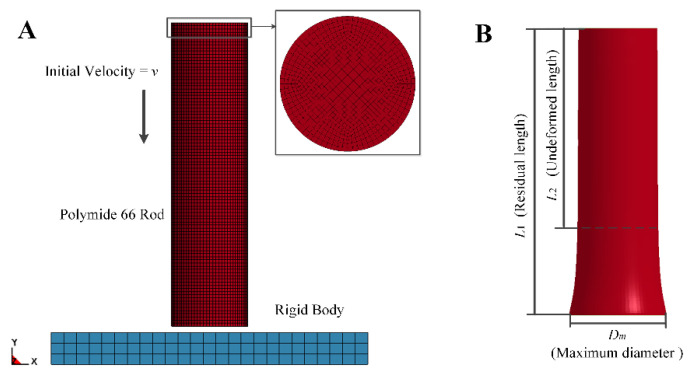
(**A**) A 3-D simulation model of Taylor impact test; (**B**) Three key dimensions of post-test bar.

A three-dimensional geometry model was established as shown in Figure 7. There were the same impact velocity and cylinder sizes between the simulation model and experimental specimen. The element size of cylindrical specimen was set to 0.03 mm, and the final selected mesh of cylindrical specimen consists of 320,000 SOLID 164 elements. Since the target was viewed as rigid body, the element size of target, including 1875 SOLID 164 elements, was chosen as 0.2 mm to reduce the computations. During simulations, all edges of the target are fixed and the cylindrical specimen possessing initial velocity was impacted on central point of target. A surface-to-surface contact model was employed to define the contact behaviour between the cylindrical specimen and the target.

### 4.3. Results and Discussion

**Figure 8:**
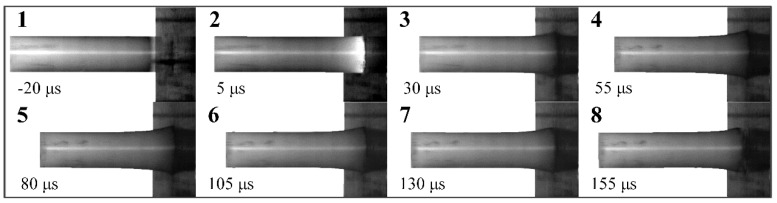
High-speed photographs of Taylor impact test at 104 m/s.

The process of a PA66 rod impacting on a target at 104 m/s was recorded by high-speed camera and shown in Figure 8. It is found that the collision results in formation of a mushroom head at the impact end, which enlarges along radial direction and the plastic zone expend to back end as the event progresses. The rod continues to compress in a plastic manner for about 80 μs, and then the rod begins to rebound. During the rebound stage, the rod still contacted with target, and the rod shows some elastic recovery. After impact, the post-test rod was recovered and measured. Residual length, deformation area length and maximum diameter are 47.06 mm, 13.56 mm, and 13.04 mm, respectively, which are listed in Table 3.

**Figure 9:**
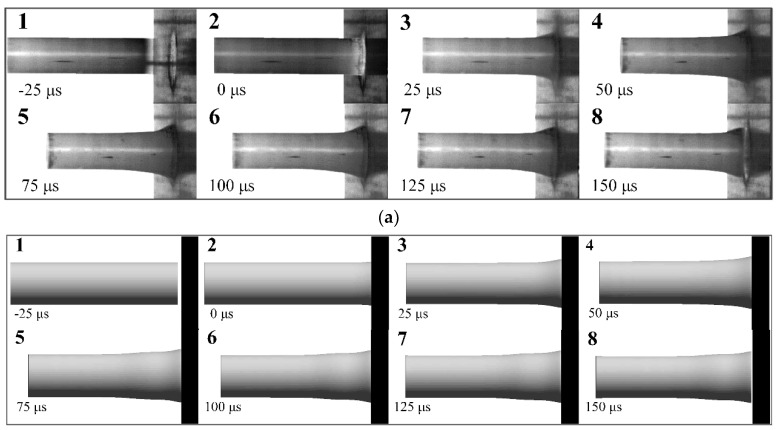
Taylor test at 128 m/s: (**a**) high-speed photographs; (**b**) deformation predictions using 3D simulation.

Taylor impact tests were also performed to investigate the dynamic response of PA66 material at higher velocity. A set of high-speed images are displayed in Figure 9a, where a PA66 rod impacts the rigid target at 128 m/s. Similar to the test with an impact velocity of 104 m/s, a mushroom head at the impact end appeared after impact. The rod sustains to heavily deform during 80 μs, and then begins to rebound. By comparing the deformation processes of the PA66 rod at 104 m/s and 128 m/s, the mushroom head and whole length of the rod display much wider deformation and an increased overall shortening, which indicates a larger deformation produced on the rod at 128 m/s. The three main sizes are also measured and listed in Table 3.

**Figure 10:**
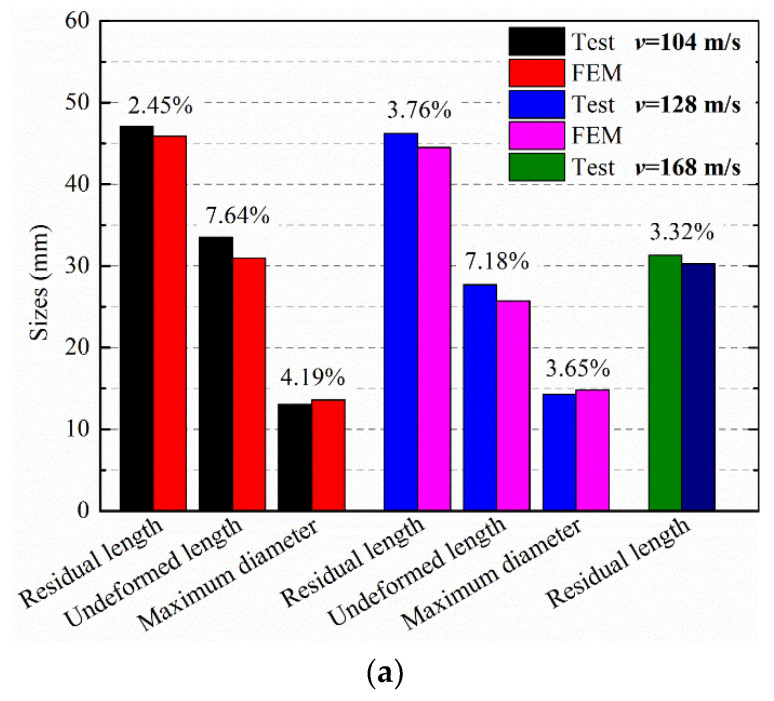

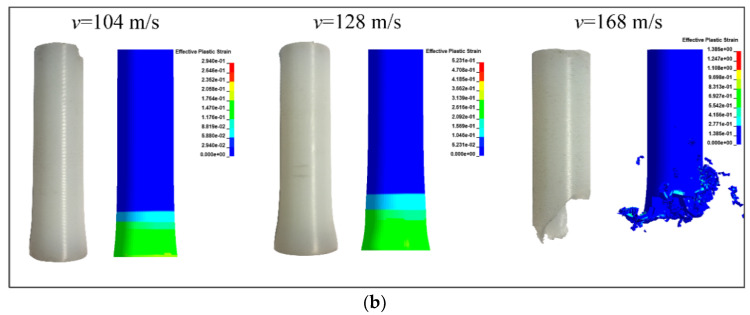
Comparison of 3D simulation results with tests: (**a**) amount of deformation (**b**) deformed shapes.

Simulations of the tests were done to predict the deformation shape of the PA66 rod and verify the accuracy of the material model. The pictures of Figure 9b show the rod geometry prior to impact and a sequence of predicted shapes at time intervals corresponding to the high-speed photographs at velocity of 128 m/s. As is observed, the deformation pattern of the rod (shown in Figure 9b) is similar to that result of the test at 128 m/s. The detailed deformation and features such as upsetting of the impact head, plastic zone extension and elastic recovery are accurately reproduced in this simulation. The residual rods (a: 104 m/s and b: 128 m/s) of the test and simulation results are compared and shown in Figure 10. Combined with geometrical dimensions of the rods (post-test and simulation) shown in Figure 10a, the errors of residual length and maximum diameter between experiments and simulations are less than 5%, and the errors of undeformed length is also under 8%. Therefore, the simulation results are in good agreement with data obtained from two sets of tests, and the simulation model is able to estimate the dynamic response accurately, including the residual length, the undeformed length, the maximum diameter and the shape of the mushroom head.

**Figure 11:**
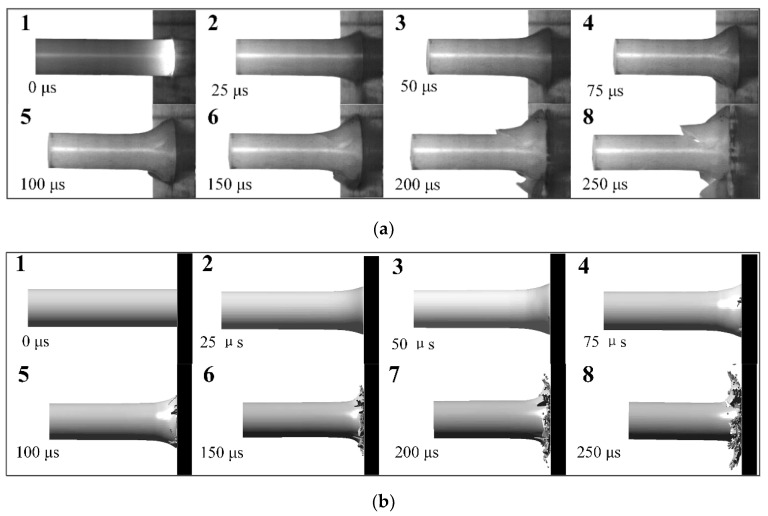
Taylor test at 168 m/s: (**a**) high-speed photographs; (**b**) deformation predictions using 3D simulation.

To analyse the dynamic response of PA66 material at higher impact speed, Figure 11a displays the high-speed images of the Taylor impact test conducted at 168 m/s. These pictures indicate that the deformation pattern is the same as previous tests prior to about 70 μs. However, the deformation of the mushroom head on radial direction abruptly increases at 75 μs after impact, since the failure arriving at the tensile limit occurs on the mushroom head of the rod. With the progress of the event, the rod begins to rebound at about 100 μs. Besides, shear and tensile cracks produce and expend along the shear mushroom head during its rebound, which are clearly observed at approximate 150 μs. Its evolution processes are exhibited in frame 5–8.

The higher velocity test was also numerically simulated to evaluate the deformation conditions and failure mode. To simulate the failure of the rod impacting target, an erosion criterion which LS-DYNA owns was used by adding keywords: MAT_ADD_EROSION. Combined with experimental results, maximum principal strain and shear strain criterions were adopted to control the failure. The evolution of the Taylor impact test and simulation is shown in Figure 11. It is found that the rod begins to fail at 75 μs, which (initial stage) agrees with the experimental phenomena. As the collision proceeds, the failure and compression of the rod aggravate. Nevertheless, the rod continues compressing to 200 μs, instead of showing signs of a rebound of the rod at 100 μs. The second stage is different from the experimental phenomena. Since the failure criterions adopted in the simulation leads elements to be deleted when the rod exceeds the failure criterions, the duration of compression is longer than that of the test. However, the failure mode and the residual rod shape (shown in Figure 10b) of the simulation are similar to these of the tests, and the error of residual length plotted in Figure 10a between simulation and test is under 3.5%. Therefore, the failure of the PA66 material is able to be roughly simulated and predicted by using maximum principal strain and shear strain criterions.

## 5. Conclusions

Based on the mechanical properties of PA66 under complex loading conditions, a Drucker Prager yield criterion was employed to describe the mechanical response. A one-dimensional constitutive model, which contains two parts (a viscoelastic and a viscoplastic regime), was introduced and converted into three dimensions. Then it was implemented into a LS-DYNA software, which was used to predict the dynamic response of PA66 under Taylor impact conditions, whose corresponding tests were conducted by gas gun and recorded by high-speed camera. By contrasting the simulation results and these of the corresponding tests, the deformed shapes including the residual length, the maximum diameter and the shape of the mushroom head of the PA66 bars were found to be similar to these obtained from the tests, which verified the accuracy of the three-dimensional constitutive model, and proved that the model was able to be applied to high-rate impact loading conditions.

## Figures and Tables

**Table 1:** Yield criterion formulas.

Number	Formula	References	Polymer
R1	$\frac{1}{2}\max\left(\sigma_{i}-\sigma_{j}\right)=k_{T}-\frac{\mu_{T}}{3}I_{1}$	Bowden and Jukes (1972)	PS
R2	$\frac{\sqrt{6J_{2}}}{3}=k_{M}-\frac{\mu_{M}}{3}I_{1}$	Bowden and Jukes (1972)	PMMA
R3	$3J_{2}+\left(C-T\right)I_{1}=CT$	Raghava et al. (1973)	PC, PVC
R4	$\sqrt{J_{2}}=\sum_{i=0}^{N}\alpha_{i}{\left(I_{1}\right)}^{i}$	Silano et al. (1974) Pae (1977)	PP, POM
R5	$\frac{3J_{2}}{T}\left(1-\frac{27}{32}\frac{J_{3}^{2}}{J_{2}^{3}}\right)+\frac{7\left(m-1\right)}{8}I_{1}-\frac{7}{8}mT=0$	Ghorbel (2008)	PMMA, PC, PS
R6	$\sqrt{J_{2}}=\alpha_{0}^{*}{\left(\frac{\dot{\varepsilon }}{{\dot{\varepsilon }}^{*}}\right)}^{\beta_{0}}+\sum_{i=0}^{N}\alpha_{i}{\left(I_{1}\right)}^{i}$	Farrokh (2010)	Nylon 101

**Table 2:** Parameters for constitutive model of PA66.

$E_{0}$ (MPa)	α	β	${\dot{\varepsilon }}_{0}$ s^−1^	p (MPa)	q	$\sigma_{ds}$ (MPa)	A	B	γ	m	n	$\alpha_{0}$	$\alpha_{1}$
2300	0.665	0.175	1	3.852	1.790	90	0.51	2665.47	1.42	0.25	0.28	40.1	−0.1

**Table 3:** Specific conditions of tests and simulations for several Taylor impact.

Test Number	*v* (m/s)	*L* (mm)	*D* (mm)	*L_t_*_1_ (mm)	*L_t_*_2_ (mm)	*D_tm_* (mm)	*L_s_*_1_ (mm)	*L_s_*_2_ (mm)	*D_sm_* (mm)
1	104	48.04	11.70	47.06	33.50	13.04	45.90	30.94	13.61
2	128	48.01	11.88	46.24	27.70	14.26	44.50	25.71	14.80
3	168	48.01	11.88	31.32	-	-	-	30.28	-

Note: *v*, *L*, *D*, *L_t_*_1*a*_, *L_t_*_2_, *D_tm_*, *L_s_*_1_, *L_s_*_2_ and *D_sm_* are the impact velocity, original length, original diameter, final length, maximum diameter, simulation length and simulation diameter of the bar, respectively.
